# Prognostic Serum Biomarkers of Inflammaging in Patients Undergoing Emergency Laparotomy

**DOI:** 10.3390/jpm13050769

**Published:** 2023-04-29

**Authors:** Michael George, Rajarshi Mukherjee

**Affiliations:** 1 Liverpool EmerGenT Academy, Department of Emergency General and Major Trauma Surgery, Aintree University Hospital, Liverpool University Hospitals NHS Foundation Trust, Lower Lane, Liverpool L9 7AL, UK; 2 Institute of Systems, Molecular & Integrative Biology, University of Liverpool, Biosciences Building, Crown Street, Liverpool L69 7BE, UK

**Keywords:** frailty, older adult, inflammaging, inflammation, biomarkers, prognosis, outcomes, emergency, laparotomy, surgery

## Abstract

Surgeons are increasingly faced with an ageing and frail patient population. There is a significant absence of biomarkers capable of risk stratifying patients undergoing emergency laparotomy. Inflammaging describes a state of chronic inflammation associated with ageing and frailty that may predict worse outcomes after surgery. This retrospective observational study evaluated pre-morbid inflammatory markers in the prognostication of older adult patients undergoing emergency laparotomy. Patients aged ≥65 years undergoing surgery between 1 April 2017 and 1 April 2022 were identified. Pre-admission and acute C-reactive protein (CRP), erythrocyte sedimentation rate (ESR), total white cell count (WCC), neutrophil count (NC) and lymphocyte count (LC) datapoints were captured. Pre-operative risk stratification scores and post-operative outcomes were recorded using the National Emergency Laparotomy Audit (NELA) database. A cohort of 196 patients was included: 57.7% were female, median age 74.5 years. High risk (NELA risk of mortality ≥ 5%) and frail (clinical frailty scale ≥ 4) patients experienced a significantly longer hospital and critical care stay (*p* < 0.05). Pre-admission ESR ≥ 16 and LC ≥ 4.1 were significantly associated with a longer critical care stay (*p* < 0.05); no statistical significance was observed with CRP, WCC and NC in predicting adverse outcomes. We found that an elevated pre-morbid ESR and LC identifies a potential inflammaging cohort that demonstrates worse outcomes following emergency laparotomy. The prognostication of older adult surgical patients remains a challenge and represents an area of research deserving of future attention.

## 1. Introduction

Surgeons are increasingly faced with an ageing and frail patient population, leading to challenging risk-benefit discussions in emergency case selection. Frailty may be defined as “a clinically recognisable state of increased vulnerability resulting from ageing-associated decline in reserve and function across multiple systems, resulting in an impaired ability to cope with acute physiological stress” [1]. Frailty represents a growing area of interest within surgical clinical practice and research. There is a significant absence of biomarkers capable of risk stratifying patients undergoing emergency laparotomy.

Emergency laparotomy is a highly invasive surgical approach most commonly reserved for cases of acute clinical instability or failed minimally invasive management. The combination of a pressurised environment, strained hospital resources and acutely unwell surgical candidate risk increases in morbidity and mortality. As a consequence, emergency laparotomy may be associated with a prolonged and complicated recovery post-operatively, as well as the risk of adverse outcomes in the interim; the likelihood of such prospects is increased in frail patients due to their reduced physiological reserve [2,3]. Case selection for emergency laparotomy therefore presents a clinical challenge. It is imperative that this at-risk cohort is identified in a timely manner in the acute setting to allow for effective, individualised counselling of the risks and benefits of surgery prior to invasive investigation and management.

At present, patients in the United Kingdom undergo frailty assessment and risk stratification prior to emergency laparotomy using both the Clinical Frailty Scale (CFS) and the National Emergency Laparotomy Audit (NELA) risk of morbidity and mortality, which incorporates the American Society of Anaesthesiologist’s Score (ASA). These clinical composite scoring systems utilise descriptive parameters such as baseline functional status and co-morbidities to generate a numerical output of risk expressed as a percentage [4,5,6]. These techniques require a comprehensive clinical history which may not be available in the emergency setting and which is subjective in nature, risking the under- or over-estimation of morbidity and mortality in certain patient groups [7].

Inflammaging is a novel concept that describes a persistent state of chronic inflammation, independent of acute illness or injury, that is associated with physiological ageing and frailty [8]. Inflammaging patients have previously demonstrated poorer outcomes following acute illness with sepsis and cardiovascular disease [8,9,10]; these findings may correlate with an adverse outcome in the post-operative period following emergency laparotomy given its significant physiological burden. Existing blood-based biomarkers, such as C-reactive protein (CRP) and erythrocyte sedimentation rate (ESR), provide rapidly accessible and cost-effective methods of detecting inflammation. The diagnostic and surveillance utility of serological inflammatory markers are widely accepted within modern-day clinical practice. Previous studies suggest that pre- and post-operative inflammatory markers may be capable of predicting adverse outcomes in specific surgical cases, such as emergency laparotomy for visceral perforation [11] and elective colorectal cancer resection [12]. Prognostication using peri-operative blood tests primarily evaluates the physiological burden of disease and/or surgery. The association between inflammaging, clinical frailty and post-operative outcome is less well established. Detection of clinical frailty using inflammaging biomarkers has the potential to overcome the raised limitations of clinical composite scoring systems by applying quantitative objective diagnostic criteria utilising readily available serological data.

This retrospective observational study aimed to identify a cohort of inflammaging patients undergoing emergency laparotomy and compare their post-operative outcomes with a non-inflammaging group.

## 2. Materials and Methods

### 2.1. Participant Selection

**Figure 1:**
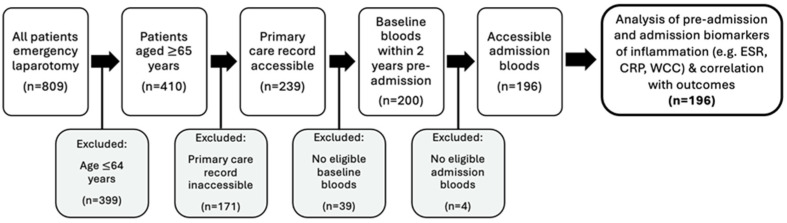
Process for observational test cohort selection and analysis based on the application of the pre-determined inclusion-exclusion criteria.

Potential study participants were identified using the NELA database. Inclusion–exclusion criteria were defined in order to capture the relevant patient cohort. The inclusion criteria identified patients aged over 65 years old undergoing emergency laparotomy within Liverpool University Hospitals NHS Foundation Trust between 1 April 2017 and 1 April 2022. The exclusion criteria identified patients aged under 65 years old, those without accessible clinical records and those without eligible serological datapoints. The process for observational test cohort selection and analysis throughout the study is summarised in Figure 1.

Participant demographic data were collected using the NELA database; parameters of interest included patient age, biological sex, past medical and surgical history, regular medications, clinical features and diagnosis.

### 2.2. Data Collection and Handling

Biomarker data were sought at two timepoints: (i) baseline serology was defined as routine monitoring blood tests conducted in primary care up to two years prior to admission (excluding datapoints within two weeks of admission); (ii) admission serology was defined as the first-ordered blood tests on arrival in hospital during the index admission. The captured serological inflammatory marker parameters included CRP, ESR, total white cell count (WCC), neutrophil count (NC) and lymphocyte count (LC). Patient clinical records were scrutinised, and data were collected contemporaneously. Baseline datapoints included blood tests performed at general health check-ups and monitoring of chronic conditions; blood tests performed during a period of acute illness were identified on manual review of the primary care records and excluded from analysis. Baseline and acute admission data were compared on an individual case basis in order to further differentiate the two timepoints. In the instance of multiple datapoints within the pre-admission period, the latest eligible value was inputted for analysis. The following in-house hospital laboratory reference ranges were applied to facilitate data interpretation and differentiate normal from abnormal test results: CRP < 5 mg/L, ESR < 16 mm/h, WCC 4.5–11.0 × 10^9^/L, NC 1.5–7.5 × 10^9^/L, LC 1.0–4.0 × 10^9^/L. Patients with elevated baseline inflammatory marker values were assigned to the inflammaging group, whilst the non-inflammaging cohort were identified with baseline inflammatory marker values within the normal range.

Established risk stratification techniques including NELA risk of mortality and CFS score were evaluated as the reference standard within the test cohort. The post-operative outcomes captured included duration of total hospital stay and duration of critical care stay. Data were also collected on admission mortality and rate of return to theatre; however, these outcomes did not undergo analysis as the comparisons lacked statistical power due to the low observation rate, as discussed in the study limitations.

Access to the relevant data was granted by Liverpool University Hospitals NHS Foundation Trust and the NELA database. Baseline biomarker data were captured using E-Exchange, a computer system that interfaces patient primary and secondary care clinical records. Acute admission biomarker data were collated using ICE, a computer system facilitating the requesting and reporting of clinical investigations within secondary care; in cases of non-accessible computerised data, the hand-written clinical notes were consulted. Pre-operative risk stratification scores and post-operative outcome data were accessed using the NELA database and consolidated using the clinical notes. The collected data were stored in spreadsheet format using Microsoft Excel 2018 [13]. Patients were anonymised and assigned a unique study identifier.

### 2.3. Statistical Analysis

Statistical analysis was facilitated by the Statistical Package for the Social Sciences (SPSS, Version 26) [14]. The Shapiro–Wilk test was applied to evaluate the distribution of data using a hypothesis of normality, whilst Levene’s test was applied to assess for equality of variance between comparison groups. Parametric statistical analysis utilised *t*-testing, with mean and standard deviation acting as measures of data spread; non-parametric statistical analysis utilised Mann–Whitney testing, with the median and interquartile range acting as measures of data spread. The conventional *p*-value threshold of 0.05 was applied to determine statistical significance.

### 2.4. Ethical Statement

This study was performed in line with the principles of the Declaration of Helsinki. The study was designed as an audit, and thus did not require ethics committee approval or the obtaining of informed consent from study participants. The data used by the researchers were anonymised and all aspects of the study were conducted in an ethical manner.

## 3. Results

### 3.1. Participant Characteristics

A total of 196 patients were included for analysis: 57.7% were female and the median age was 74.5 years (interquartile range [IQR] 12). The most commonly presenting features were abdominal pain, nausea, vomiting and constipation; 58.2% of patients presented with two or more of the listed symptoms. The most frequently observed diagnoses leading to emergency laparotomy were small bowel obstruction, visceral perforation and complicated hernia. The cohort exhibited a varied past medical, surgical and medication history. Within the study cohort, the median total hospital and critical care stay measured 15 days (IQR 15) and 1 day (IQR 3), respectively. The participant characteristics are summarised in Table 1.

### 3.2. Clinical Frailty Scale, NELA Risk of Mortality and Actual Mortality

**Figure 2:**
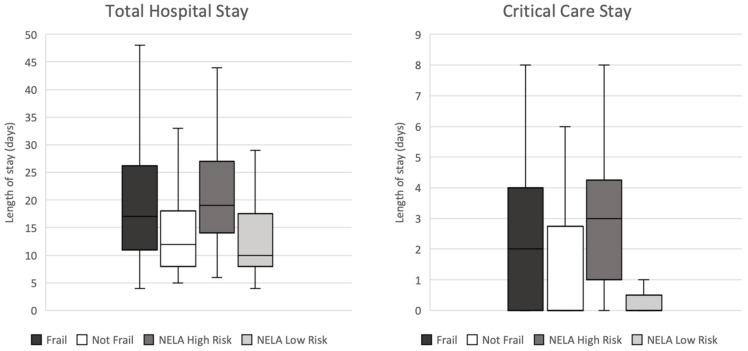
Box and whisker plots illustrating the association between CFS score and NELA risk of mortality, and length of total hospital and critical care stay: frail, CFS ≥ 4; not frail, CFS ≤ 3; NELA high risk, risk of mortality ≥ 5%; NELA low risk, risk of mortality < 5%.

The CFS and NELA risk of mortality were first evaluated to benchmark the current standards of care in the identification and risk stratification of frail patients within our specific test cohort. Frail patients, defined as CFS ≥ 4, experienced a significantly prolonged stay in critical care (*p* = 0.003, median 2 days compared with 0 days) and hospital (*p* = 0.004, median 17 days compared with 12 days) on Mann–Whitney testing. Furthermore, high risk patients, defined as NELA risk of mortality ≥ 5%, also exhibited a significantly lengthened stay in critical care (*p* < 0.001, median 3 days compared with 0 days) and hospital (*p* < 0.001, median 19 days compared with 10 days) on Mann–Whitney testing. The results of these analyses are visualised in Figure 2.

The overall admission mortality rate was 0.5% (*n* = 1) within the study cohort (*n* = 196). The deceased patient was identified as high risk (NELA risk of morality = 20.3%) prior to undergoing emergency repair of perforation secondary to peptic ulcer disease. In this isolated case, review of the pre-admission serology demonstrated LC 3.2 and no eligible ESR data, meaning they were assigned to the non-inflammaging group in subsequent analyses. The adverse outcome observed in this individual case did not correlate with an elevated LC.

### 3.3. Erythrocyte Sedimentation Rate

**Figure 3:**
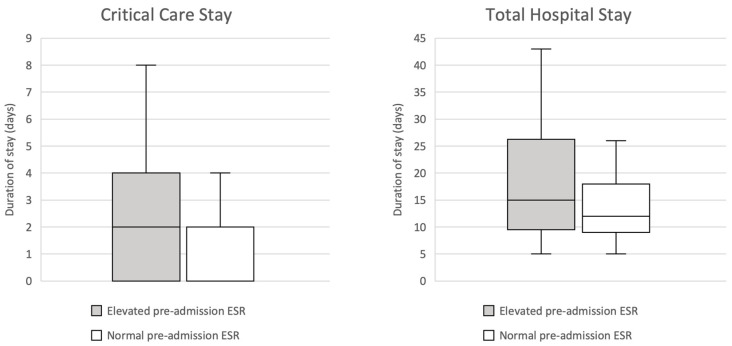
Box and whisker plots illustrating the association between pre-admission ESR and length of critical care (*p* = 0.012) and total hospital stay (*p* = 0.137): elevated, pre-admission ESR ≥ 16; non-elevated, pre-admission ESR ≤ 15.

An elevated ESR, defined as ≥16 using the pre-determined reference range, was associated with a significantly prolonged critical care admission on Mann–Whitney testing (*p* = 0.012, median 2 days compared with 0 days). It also demonstrated an observed trend with prolonged total hospital stay, although this analysis did not achieve statistical significance (*p* = 0.137, median 15 days compared with 12 days). These findings are illustrated in Figure 3.

### 3.4. Lymphocyte Count

**Figure 4:**
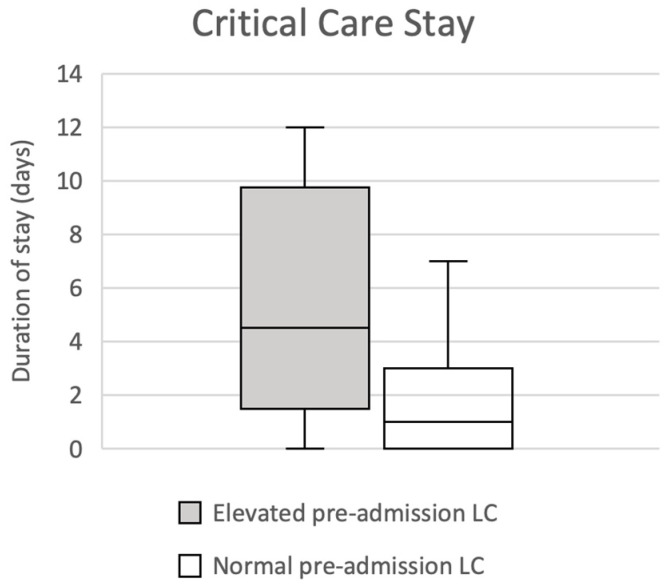
Box and whisker plots illustrating the association between pre-admission LC and length of critical care stay (*p* = 0.035): elevated, pre-admission LC ≥ 4.1; non-elevated, pre-admission LC ≤ 4.0.

Mann–Whitney testing identified a statistically significant association between an elevated LC, defined as ≥4.1 using the pre-determined reference range, and extended critical care stay (*p* = 0.035, median 4.5 days compared with 1 day); this has been illustrated in Figure 4. No correlation of significance was observed on analysis of total hospital stay duration in this group (*p* = 0.608, median 12.5 days compared with 15 days).

### 3.5. C-Reaction Protein, Total White Cell Count and Neutrophil Count

Analysis of the remaining serological parameters including CRP, WCC and NC did not demonstrate any association with length of total hospital and critical care stay of statistical significance. The results of these analyses are summarised in Table 2.

## 4. Discussion

### 4.1. Overview

This pragmatic retrospective observational study evaluated the clinical potential of pre-admission serological biomarkers of inflammation in the identification and prognostication of frail patients undergoing emergency laparotomy. The CFS and NELA risk of mortality represented the current standards of care within the United Kingdom in this study; high risk and frail patients, as determined using these techniques, experienced significantly prolonged total hospital and critical care stays. An elevated pre-admission ESR and LC were significantly associated with prolonged critical care admission; these biomarkers may have the potential to support the pre-operative risk stratification of older adult and frail surgical patients in the future.

### 4.2. Clinical Relevance and Future Application

Emergency laparotomy is a highly invasive surgical technique that requires physiological resilience of the surgical candidate for satisfactory surgical outcome and recovery. The risks associated with emergency laparotomy, such as wound and secondary infections, long-term morbidity and mortality are widely recognised [15]. Older adults and frail patients represent a cohort with reduced physiological reserve, impairing their ability to tolerate the intense burden of emergency laparotomy; as a result, the likelihood and severity of adverse outcomes is significantly increased [7]. Effective surgical case selection in this group requires critical evaluation of the potential risks and benefits of surgery and is central to their management.

Pre-operative risk stratification currently utilises clinical composite scoring systems such as the NELA risk of morbidity and mortality to support and facilitate this decision-making process. Clinical composite scoring systems can be time consuming, require a comprehensive clinical history and are subjective in nature, bringing their utility in the emergency setting into question. Inflammaging has the potential to overcome these challenges by offering a quantitative approach to the identification of frail patients using readily accessible, non-invasive serological data. Given that inflammaging patients have previously been shown to experience worse outcomes following periods of acute illness, it may be hypothesised that they are also at increased risk of adverse outcomes following surgery. The findings reported in the present study are supportive of this notion: an elevated pre-admission ESR and LC identified a group of inflammaging patients that did indeed experience worse outcomes with prolonged critical care admission.

With further research and larger prospective studies, inflammaging may demonstrate the potential to assist the pre-operative risk stratification of older adult surgical patients. The application of a validated inflammaging biomarker panel would support clinicians in identifying clinically frail older adult patients in a timely manner during periods of acute illness. This would in turn facilitate an individualised approach to risk-benefit discussions prior to emergency surgery in this at-risk cohort. In practical terms based on the present widely available technology, patients would be required to attend semi-regular blood tests in the community in order to determine their inflammaging status. Whilst this is feasible, its introduction at a population level may pose logistical and financial challenges, which could be overcome with further research to optimise the exact timing and frequency of such blood tests.

### 4.3. C-Reactive Protein and Erythrocyte Sedimentation Rate

The CRP and ESR are often regarded as the most widely recognised and commonly requested serological inflammatory markers. These two assays are capable of detecting and quantifying active inflammation within the body, in turn supporting clinicians with their clinical decision-making [16]. Interestingly, the present study reports patients with an elevated ESR were associated with significantly worse post-operative outcomes, whilst those with an elevated CRP were not. One possible explanation for this lies within the physiology underpinning the two assays.

CRP synthesis in the liver is stimulated by pro-inflammatory inflammatory cytokines such as interleukin-6, interleukin-1β and tumour necrosis factor α [17]. CRP is a dynamic biomarker that is highly sensitive to slight variations in inflammatory states, translating to data values that increase in accordance with increasing severity of inflammation [18,19]. The rapid ascent and fall of CRP, in proportionate response to inflammation, make it a popular assay in the diagnosis and surveillance of acute inflammatory disorders [20].

ESR, as its name suggests, measures the sedimentation rate of erythrocytes within plasma. This process is dictated by the aggregation of red blood cells forming rouleaux, and is mediated by acute phase reactants including fibrinogen [21]. Fibrinogen is a pro-thrombotic protein synthesised in the liver that, in its capacity as an acute phase reactant, is slow-reacting and insensitive to mildly differing inflammatory states [22].

It is therefore plausible that, in states of established chronic inflammation, ESR has the potential to offer a more reliable insight into an individual’s chronic health status, whilst CRP risks labile readings that may over-represent short-term variations. Additional research is recommended to further evaluate the association between ESR and clinical frailty and its potential clinical utility in surgical risk stratification.

### 4.4. Inflammaging in Surgery

Hasselgager et al. investigated serological immune parameters such as CRP, interleukin-6, interleukin-10, interferon-γ induced protein 10 kDa, tumour necrosis factor α and soluble urokinase plasminogen receptor activator in the prognostication of laparotomy patients [23]. This pilot study of 100 patients reported a regression model that utilised these biomarkers in combination with patient demographics and clinical composite scoring systems (ASA physical status and Eastern Cooperative Oncology Performance Status) demonstrating improved predictive accuracy for major complications and mortality [23]. Although these findings evidence the clinical potential of immunological serology in pre-operative risk stratification, they also highlight two persisting challenges: (i) the need for non-routine, selective investigations required for application of the reported model; (ii) the reliance on clinical composite scoring systems as an adjunct to improve predictive performance.

Simpson et al. evaluated the neutrophil/lymphocyte ratio (NLR) and CRP/albumin ratio (CAR) as predictors of mortality in 136 older adult patients undergoing emergency laparotomy [11]. An elevated pre-operative NLR was significantly associated with inpatient, 30-day and 90-day mortality in patients with visceral perforation; however, it demonstrated no significance in cases without peritoneal contamination [11]. No association was observed between CAR and post-operative morbidity and mortality [11].

Further research, published by Vaughan-Shaw et al., found NLR to be an independent predictor of mortality at 30-day, 6-month and 12-month time points in patients undergoing emergency abdominal surgery [24]; this study included a wide variety of emergency surgical cases and procedures, reporting laparotomy itself to also be a predictor of adverse outcome [24].

Drawing a direct comparison between the present study and the existing literature is challenging given the discrepancies in methodological approach (patient group, surgical procedure and serology collected). However, the potential clinical utility of inflammaging remains evident and highlights the need for further research to harmonise research methodology.

### 4.5. Limitations and Further Considerations

This study met its pre-determined aim to investigate serum biomarkers of inflammation in the prognostication of older adult patients undergoing emergency laparotomy. A representative cohort of real-world patients were identified and underwent retrospective analysis. Inflammaging demonstrated the potential to influence future clinical practice in risk-stratifying patients prior to emergency laparotomy. However, given its single-centre design, the findings reported in this study would benefit from external validation.

The outcomes reported in this research included duration of hospital and critical care admission. Additional prognostic outcomes of potential interest might include rates of mortality (during admission, 30-day, 3-month, 6-month and 12-month intervals) and post-operative complications. A further consideration is that this study provides only a snapshot insight into the index admission without scope for follow-up. In light of this and the low observation rate within the included cohort, mortality and complication data did not undergo analysis; as a result, duration of hospital and critical care stay acted as a proxy for post-operative outcome. Future research with larger and independent data may address this limitation and build upon the present study.

Furthermore, length of hospital and critical care stay may be influenced by several factors which may be considered to fall into one of two categories: clinical and non-clinical. On review of the literature, a study of 215,724 patients published by Achanta et al. reported that most variation observed in length of stay amongst emergency general surgery admissions is due to non-clinical factors such as logistical delays [25]. Additionally, Ward et al. reported that non-surgical variables, including performance in activities of daily living (assessed using the Barthel index), housing status and social support, influence length of stay [26]. Considering other surgical specialities and the elective surgery setting, factors such as comorbidities, malnutrition and abnormal blood test parameters have all been reported to influence length of hospital and critical care stay [27,28]; however, no consensus exists on the key factors impacting length of stay, particularly within a representative population, as this is likely to be varied and heterogeneous.

This exploratory study recruited a five-year cohort of real-world patients to evaluate potential prognostic biomarkers in a manner that would represent their future use in clinical practice. As such, patient demographic data including co-morbidities and medication history were collected for descriptive purposes but not adjusted for within the analysis.

## 5. Conclusions

This study evaluated readily available serological markers of inflammation as potential prognostic biomarkers in patients undergoing emergency laparotomy. We found that an elevated pre-admission ESR and LC identifies a cohort of older adult patients with inflammaging that experience adverse outcomes following emergency laparotomy. Detection of frailty using inflammaging biomarkers may have the potential to support surgical case selection and promote improved personalised patient care. The study crucially highlights that the molecular identification and prognostication of biologically frail older adult surgical patients remains a challenge and represents an area of research deserving of future attention; such research may build upon the foundations of this work and look to develop new composite clinical scores or explore novel biochemical and genetic assays.

## Figures and Tables

**Table 1:** Summary of participant characteristics.

Characteristic	Frequency (Percentage of Total)
Gender	
Male	83 (42.3%)
Female	113 (57.7%)
Age (years)	
65–74	98 (50.0%)
75–84	73 (37.2%)
85–94	25 (12.8%)
Primary clinical feature(s)	
Abdominal pain	148 (75.5%)
Nausea +/− vomiting	72 (36.7%)
Constipation	47 (21.4%)
Non-specifically unwell	15 (7.7%)
Abdominal distension	12 (6.1%)
Diarrhoea	10 (5.1%)
Haematochezia or melaena	6 (3.1%)
Diagnosis	
Small bowel obstruction	84 (41.8%)
Perforation	34 (17.3%)
Complicated hernia	29 (14.8%)
Large bowel obstruction	23 (11.7%)
Ischaemic colitis	6 (3.1%)
Diverticular disease	6 (3.1%)
Unknown	5 (2.6%)
Volvulus	2 (1.0%)
Appendicitis	2 (1.0%)
Anastomotic leak (following previous surgery)	2 (1.0%)
Colorectal cancer	2 (1.0%)
Bile duct injury (following previous surgery)	1 (0.5%)
Past medical history	
Chronic respiratory disease (COPD or asthma)	52 (26.5%)
Hypertension	101 (51.5%)
Ischaemic heart disease	31 (15.8%)
Atrial fibrillation	28 (14.3%)
Inflammatory bowel disease	9 (4.6%)
Diabetes mellitus	35 (17.9%)
Chronic kidney disease	21 (10.7%)
Cerebrovascular disease (stroke or TIA)	23 (11.7%)

**Table 2:** Summarised statistical output following analysis of CRP, WCC and NC versus total hospital and critical care stay with Mann–Whitney testing; all analyses failed to achieve statistical significance (*p* > 0.05).

Serological Parameter	Outcome	*p*-Value	Median (Elevated/Non-Elevated)
CRP > 5	Critical care stay	*p* = 0.638	2 days:2 days
	Total hospital stay	*p* = 0.561	17 days:17 days
WCC > 11	Critical care stay	*p* = 0.115	0 days:2 days
	Total hospital stay	*p* = 0.065	11 days:15 days
NC > 7.5	Critical care stay	*p* = 0.325	0 days:2 days
	Total hospital stay	*p* = 0.336	11 days:15 days

## Data Availability

The study data are available on reasonable request to the corresponding author.
